# Efficacy and safety of Xiaoaiping injection for liver cancer: A protocol for systematic review and meta-analysis

**DOI:** 10.1097/MD.0000000000021993

**Published:** 2020-08-28

**Authors:** Daorui Hou, Jian Xiong, Ya Li, Yahui Peng, Lu Xiong

**Affiliations:** a Department of Traditional Chinese Medicine Oncology, The First People's Hospital of Xiangtan City, Xiangtan, Hunan Province; b Department of Oncology, Guang’anmen Hospital, Beijing; c Hangzhou Lin’an TCM Hospital, Hangzhou, Zhejiang Province; d Beijing Shahe Hospital, Beijing, China.

**Keywords:** liver cancer, protocol, systematic review and meta-analysis, Xiaoaiping

## Abstract

**Background:**

Xiaoaiping injection, extracted from the Chinese herb *Marsdenia tenacissima (Roxb.) Wight et Arn*., is a broad-spectrum anti-tumor drug and has been widely used for the treatment of liver cancer in China. The aim of this study is to systematically investigate the efficacy and safety of Xiaoaiping injection for the treatment of liver cancer.

**Methods and analysis:**

Seven electronic databases including the Cochrane Library, PubMed, Excerpt Medica Database, Chinese Biomedical Literature Database, China National Knowledge Infrastructure, China Scientific Journal Database, and Wanfang Database will be systematically retrieved for data extraction from their inceptions to August 2020. Cochrane Risk of Bias tool will be used to assess the risk of bias of included studies. The RevMan 5.4 and Stata 16.0 software will be applied for statistical analyses. Statistical heterogeneity will be computed by *I*^*2*^ tests. Sensitivity analysis will be conducted to evaluate the stability of the results. The publication bias will be evaluated by funnel plots and Eggers test. The quality of evidence will be assessed by the GRADE system.

**Results:**

The results of our research will be published in a peer-reviewed journal.

**Conclusion:**

The conclusion of this study will provide helpful evidence of the effect and safety of Xiaoaiping injection for the treatment of liver cancer in clinical practice.

**OSF registration number:**

10.17605/OSF.IO/9BD6A.

## 1 Introduction

Liver cancer is one of the most common malignancies worldwide and the second most common cause of cancer-related death.^[1]^ The 5-year overall survival rates are 10% for locally advanced and 3% for metastatic disease.^[2]^ Limited progress has been made in systemic treatment for liver cancer over the past decade.^[3]^ The increase in liver cancer incidence, the undruggable nature of liver cancer mutations, and unresponsiveness of these tumors to therapy highlight the urgency to develop more effective therapeutic approaches for it.^[4–6]^

Traditional Chinese medicines (TCMs) has been effectively applied in treating malignant diseases for thoudsands of years in China and is widely used as an alternative or combined treatment for cancers.^[7–9]^ In recent years, researchers have found that many TCMs, as well as compounds extracted from certain Chinese medicines, have outstanding anti-tumor effect.^[10–13]^ Medicinal herbs and their derivative phytocompounds are being increasingly recognized as useful complementary treatments for cancer,^[14]^ and some of them have been used clinically to the treatment of liver cancer.

Xiaoaiping injection, extracted from the Chinese herb *Marsdenia tenacissima (Roxb.) Wight et Arn*., was approved by the China Food and Drug Administration (Drug Approval Number: Z10970091) for the treatment of various cancers, such as esophageal cancer,^[15]^ gastric cancer,^[16]^ lung cancer,^[17]^ and breast cancer.^[18]^ Clinical studies have reported the beneficial effects on the survival, immune modulation, and quality of life of cancer patients, when Xiaoaiping injection are used in combination with conventional therapeutic.^[15,19]^ Studies have shown that Xiaoaiping injection can inhibit the growth of liver cancer and enhance the quality of life of patients with cancers.^[20,21]^ The combination of Xiaoaiping injection and chemotherapies exerts an enhanced therapeutic effect against malignant tumors, and it does not increase the toxicity or side effects of chemotherapy.^[18,22]^

With the publication of numbers of trials and clinical studies on Xiaoaiping injection for liver cancer, there is an urgent need for a systematic review to assess the effectiveness and safety of Xiaoaiping injection in treating liver cancer. Hereby, the aim of our study is to systematically review current available randomized controlled trials (RCTs) and to objective comment the efficacy and safety of Xiaoaiping injection in the treatment of liver cancer and to provide a reference for clinical application.

## 2 Methods and analysis

This study was prospectively registered in the Open Science Framework with a DOI: 10.17605/OSF.IO/9BD6A. The protocol of our meta-analysis will be conducted followed the guideline of the Preferred Reporting Items for Systematic Review and Meta-Analysis Protocols (PRISMA-P) recommendations.^[23]^

### 2.1 Inclusion criteria

#### 2.1.1 Type of study

All RCTs that investigated the efficacy and safety of Xiaoaiping injection for the treatment of liver cancer will be included in this systematic review. Observational studies, cross-over studies, conference abstracts, animal studies, and letters will be excluded. There are no limitations on language and publication status.

#### 2.1.2 Types of participants

Any participants who are diagnosed as liver cancer will be considered for inclusion regardless their age, gender, race, condition duration, or intensity.

#### 2.1.3 Types of interventions

Interventions to be reviewed are Xiaoaiping injection alone or combinations with other interventions to treat liver cancer. When Xiaoaiping injection used as combinations with other treatments, the control group should also receive the same combination treatments.

#### 2.1.4 Types of outcomes

The primary outcomes will focus on overall survival (OS) and progression-free survival (PFS). The secondary outcomes included overall response rate (ORR), disease control rate (DCR), quality of life improved rate (QIR), and adverse events.

### 2.2 Search strategy

Seven electronic databases including the Cochrane Library, PubMed, Excerpt Medical Database, Chinese Biomedical Literature Database, China National Knowledge Infrastructure, China Scientific Journal Database, and Wanfang Database will be systematically retrieved for eligible studies from their inceptions to August 2020. Additionally, we will also search Google scholar, Baidu scholar, conference proceedings, clinical registration websites, and reference lists of associated reviews to identify grey literatures. That may avoid missing any potential studies. An example of search strategy for PubMed database was as follows, and the similar search strategies will be utilized to other electronic databases:

- #1 Search: Search: (“Liver Neoplasms”[Mesh]) OR (((((((((((((((((((((Neoplasms, Hepatic[Title/Abstract]) OR (Neoplasms, Liver[Title/Abstract])) OR (Liver Neoplasm[Title/Abstract])) OR (Neoplasm, Liver[Title/Abstract])) OR (Hepatic Neoplasms[Title/Abstract])) OR (Hepatic Neoplasm[Title/Abstract])) OR (Neoplasm, Hepatic[Title/Abstract])) OR (Cancer of Liver[Title/Abstract])) OR (Hepatocellular Cancer[Title/Abstract])) OR (Cancers, Hepatocellular[Title/Abstract])) OR (Hepatocellular Cancers[Title/Abstract])) OR (Hepatic Cancer[Title/Abstract])) OR (Cancer, Hepatic[Title/Abstract])) OR (Cancers, Hepatic[Title/Abstract])) OR (Hepatic Cancers[Title/Abstract])) OR (Liver Cancer[Title/Abstract])) OR (Cancer, Liver[Title/Abstract])) OR (Cancers, Liver[Title/Abstract])) OR (Liver Cancers[Title/Abstract])) OR (Cancer of the Liver[Title/Abstract])) OR (Cancer, Hepatocellular[Title/Abstract]))
- #2 Search: (“Marsdeniae tenacissimae extract” [Supplementary Concept]) OR (((((((Xiao-Ai-Ping[Title/Abstract]) OR (Xiaoaiping[Title/Abstract])) OR (XAP[Title/Abstract])) OR (XAPI[Title/Abstract])) OR (Marsdenia tenacissima[Title/Abstract])) OR (MTE[Title/Abstract])) OR (tongguanteng[Title/Abstract]))
- #3 Search: (((((((((randomized controlled trial[Title/Abstract]) OR RCT[Title/Abstract]) OR random[Title/Abstract]) OR randomly[Title/Abstract]) OR random allocation[Title/Abstract]) OR allocation[Title/Abstract]) OR randomized control trial[Title/Abstract]) OR controlled clinical trial[Title/Abstract]) OR clinical trial[Title/Abstract]) OR clinical study[Title/Abstract]
- #1 and #2 and #3

### 2.3 Study selection and data extraction

#### 2.3.1 Selection of studies

**Figure 1:**
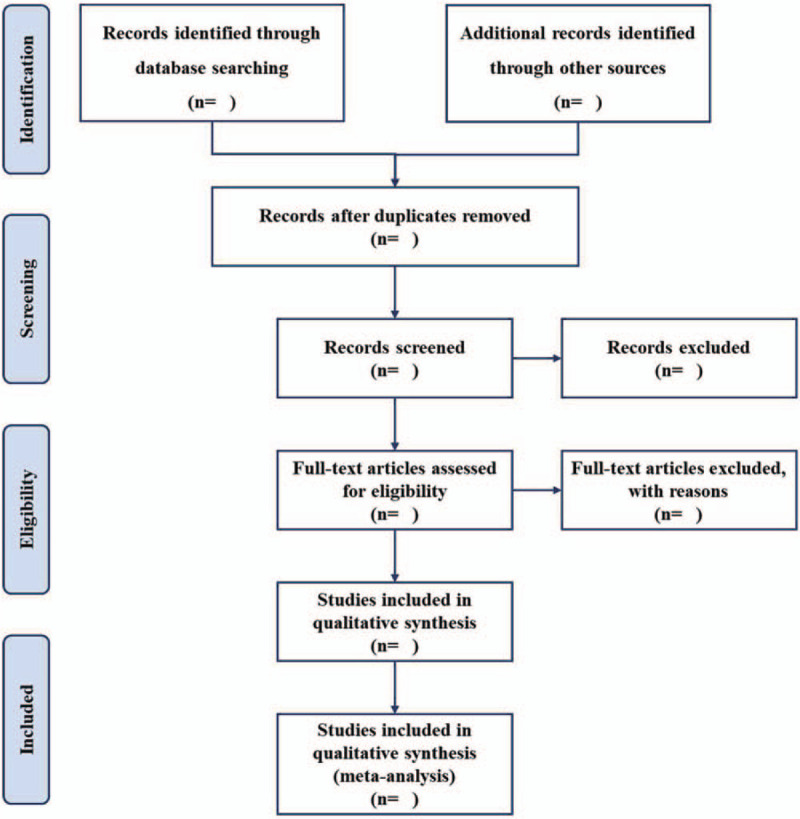
Flow chart of study selection.

EndNote X^9^ software (Clarivate Analytics, Philadelphia, USA) will be used to manage all searched results, and any duplicates will be removed.^[24]^ Two independent researchers will review the titles/abstracts of all searched studies in accordance with the inclusion and exclusion criteria. Full papers of potential studies will be reviewed if necessary. Any divergences between 2 researchers will be solved by discussion with another researcher. All excluded studies will be listed in a table with reasons. The flow chart (Fig. 1) will be used to present the whole process of study selection.

#### 2.3.2 Data extraction and management

Two researchers will independently perform data extraction according to the standardized sheet recommended by the Cochrane Handbook of Systematic Reviews of Interventions. If we identify some unclear or missing data, primary corresponding authors will be contacted to obtain it whenever possible. Any unresolved disagreements between 2 researchers will be solved by a third researcher through discussion. The data of those qualified articles will be export to Microsoft Excel, which includes basic information (registered identification, first author, authors unit, country, and publication year), research design (sample size, random sequence generation, allocation concealment, analysis of the data, processing of missing data, blinding of the participants, blinding of the outcome measurement, and blinding of the assessors), participants (disease, age, disease stage, and diagnostic criteria), details of treatment and comparison (e.g., delivery methods, dosage, and frequency), outcomes (outcome measurement), adverse events, conflicts of interest, and other essential information. Once the extraction is complete, the 2 researchers will check with each other to ensure the accuracy of the data.

#### 2.3.3 Assessment of risk of bias

Risk of bias assessment will be assessed by 2 independent researchers using the tool introduced in the Cochrane Handbook for Systematic Reviews of Interventions.^[25]^ This tool has 7 domains include random sequence generation, allocation concealment, blinding of the participants and personnel, blinding of the outcome assessments, incomplete outcome data, selective reporting, and other sources of bias. Three different grades (high risk of bias, unclear risk of bias, and low risk of bias) will be utilized to check each aspect for all included RCTs. Inconsistencies will be resolved by discussion within the group.

#### 2.3.4 Synthesis of data

RevMan 5.4 and Stata 16.0 software will be applied to carry out statistical analysis. Risk ratio (RR) or odds ratio (OR) and 95% confidence interval (CI) will be used for dichotomous outcomes. Ninety five percent CI and mean difference (MD) or standardized mean difference (SMD) will be used for continuous outcomes.

#### 2.3.5 Assessment of heterogeneity

Cochrane *X*^2^ and *I*^*2*^ tests will be conducted to assess the heterogeneity analysis between studies.^[26]^ If *P* ≥ .05 and *I*^*2*^ ≤ 50%, it suggests that no statistical heterogeneity is observed between subgroups, and the Mantel-Haenszel fixed model will be employed for meta-analysis. If *P* < .05 and *I*^*2*^ > 50%, it is considered that there is great heterogeneity between the studies, and the random effect model will be used.

#### 2.3.6 Subgroup analysis

In the case of high heterogeneity, we will conduct subgroup analysis according to the different types of studies, study characteristics, and types of intervention and comparators. The credibility of the subgroup analysis will be evaluated in term of the guidance.^[27]^ If there is a substantial heterogeneity and quantitative synthesis is not appropriate, the results will be presented in the form of tables and figures.

#### 2.3.7 Sensitivity analysis

Sensitivity analysis will be conducted to identify the stability and the robustness of the study results by removing low quality studies.

#### 2.3.8 Assessment of reporting bias

Reporting bias will be checked using funnel plot and Egger regression test if sufficient eligible trials (over 10 studies) are included.^[28,29]^*P* < .05 is considered to have publication bias.

#### 2.3.9 Grading the quality of evidence

The quality of evidence will be assessed by 2 independent researchers using the Grading of Recommendations Assessment, Development and Evaluation (GRADE), a widely used tool in evaluating the quality of assessment.^[30]^ The quality of evidence will be graded as high, moderate, low, and very low.

### 2.4 Patient and public involvement

Patient and public were not involved in this study.

### 2.5 Ethics and dissemination

This systematic review will not require ethical approval because there are no data used in our study that are linked to individual patient data. This systematic review will be disseminated through a peer-reviewed publication.

## 3 Discussion

Liver cancer has become one of the main diseases threatening human health in the 21st century.^[31]^ Xiaoaiping injection, a famous Chinese patent medicine extracted from *Marsdenia tenacissima (Roxb.) Wight et Arn*., has been widely used for the treatment of liver cancer in clinical practice in China. With an increasing number of clinical trials, it is urgent to systematically evaluate the efficacy of Xiaoaiping injection in the treatment of Hepatocellular carcinoma.^[20,21]^ In this study, we will conduct systematic review and meta-analysis to provide more evidence on the effectiveness and safety for it. The findings of this study will provide helpful evidence for clinicians in the treatment of liver cancer.

## 4 Amendments

If amendments are needed, we will update our protocol to include any changes in the whole process of research.

## Author contributions

**Conceptualization:** Daorui Hou, Lu Xiong.

**Data curation:** Daorui Hou, Jian Xiong, Yahui Peng.

**Formal analysis:** Ya Li, Yahui Peng.

**Funding acquisition:** Lu Xiong.

**Investigation:** Jian Xiong, Ya Li, Yahui Peng.

**Methodology:** Daorui Hou, Jian Xiong, Lu Xiong.

**Project administration:** Lu Xiong.

**Resources:** Daorui Hou, Jian Xiong, Yahui Peng.

**Software:** Daorui Hou, Jian Xiong, Ya Li.

**Supervision:** Lu Xiong.

**Writing – original draft:** Daorui Hou, Jian Xiong.

**Writing – review & editing:** Daorui Hou, Jian Xiong, Ya Li, Yahui Peng, Lu Xiong.
